# Appraisal of gene action for indeterminate growth in mungbean [*Vigna radiata* (L.) Wilczek]

**DOI:** 10.3389/fpls.2015.00665

**Published:** 2015-09-02

**Authors:** Javed Iqbal, Muhammad Ahsan, Muhammad Saleem, Asghar Ali

**Affiliations:** ^1^Department of Plant Breeding and Genetics, University of AgricultureFaisalabad, Pakistan; ^2^Department of Agronomy, University of AgricultureFaisalabad, Pakistan

**Keywords:** degree of indetermination, plant height, yield, harvest index, mungbean

## Abstract

In order to investigate the inheritance pattern of indeterminate growth in *Vigna radiata*, various related traits were studied. The techniques used for the purpose were generation mean and variance analyses. Narrow sense heritability estimates were also computed. Four out of fifty greengram accessions were selected during preliminary screen trial based on DDd_2_ and DDh_2_ values. Two cross combinations were made by utilizing four parents. Generation variance analysis demonstrated the engagement of additive and environmental components, with the pre-pondrance of additive gene action. Narrow sense heritability estimates (>67%) also supported the same. In generation mean analysis both cross combinations manifested non-allelic epistatic digenic interactions for the investigated traits except for plant height at first flower initiation and for seed yield per plant in one cross combination, where only additive and dominance components were important. For pyramiding the additive genes favoring determinate plant growth, higher harvest index and simultaneously purging the genes promoting twining growth habit escorted with low seed yield, any modified breeding scheme which could serve the said purpose may be opted.

## Introduction

In higher order plant species growth of the flowering stem may be either indeterminate or determinate (Weberling, 1989). The apex grows for indefinite period with the production of flowers in continuous succession in case of indeterminate plant growth (Lampang et al., 1988). While in case of determinate growth, the vegetative stage/phase terminates prior to blooming. One criterion for greengram categorization could be the plant growth habit. In legumes crops indeterminate/twinning growth habit is a natural phenomenon (Tickoo et al., 1996). The same is a serious obstacle while achieving a satisfactory crop yield during first picking. In this regard, a portion of photosynthates actually consumed while supporting the vegetative plant growth. Increase in plant height, accumulation of dry matter, including leaf area index (LAI) may be called as vegetative growth. Maximum vegetative growth prior to flowering is desirable while minimum increase in plant height after that is advantageous so as to minimize the competition among plants (Shanmugasundaram et al., 1977) with effective utilization of photosynthates for fruiting purpose. During pods development phase effective utilization of assimilates is necessary. For accomplishing the same large general combing ability effect for plant height after first flower to 90% pods maturity is mandatory (Tickoo et al., 1996). But Initial studies have revealed that determinate genotypes of some legumes gave low yield in comparison to indeterminate types (Podleœny, 2001). Apparently indeterminate growth is a desirable plant feature but the same unnecessarily prolongs the crop ripening period. From the farmer's point of view, indeterminate growth is not necessary, because extra pickings become mandatory for attaining a satisfactory crop yield. Ultimately the cost of production escalates. Indeterminate plant growth is also an obstacle toward mechanical harvesting of the crop. In this scenario, development of mungbean lines with no or minimum twining growth at the onset of blooming phase with maximum seed yield and higher harvest index will be the ultimate choice of the farmer. For accomplishing the said task, an understanding of the genetic mechanisms controlling the inheritance of plant height at various reproductive stages is mandatory. This may aid in devising a work oriented breeding strategy for curtailing the indeterminate growth in mungbean. Keeping in view the importance of determinate type of plant growth and attributing traits, present study was designed to assess the extent of gene/genes governing the inheritance of said parameters.

## Materials and methods

### Selection of parents for genetic studies

The research studies pertaining to the mode of inheritance of plant height, degree of indeterminations of plant height and other growth related traits were conducted at the experimental area of the Department of Plant Breeding and Genetics, University of Agriculture, Faisalabad, Pakistan during 2009–2010. Fifty diverse mungbean lines including those which were characterized for their determinate/indeterminate growth habit were obtained from various national research institutes (Table 1). For screening the appropriate parents, a triplicate randomized complete block design was exercised. Row length was maintained at 4 m, while 30 cm distance was kept between rows. A recommended plant spacing (10 cm) was followed. For digging the holes at the marked areas of the soil a hand-held dibbler was used. Manually added 2–3 seeds per hole. One week after germination thinning was performed. One vigorous seedling was kept per hole. Recommended agronomic and plant protection measures were adopted. Five random and guarded plants from each genotype within a replication were selected for recording the following data:

**Table 1:** **Mungbean [***Vigna radiata*** (L.) Wilczek] accessions tested**.

**Code**	**Genotype**	**Source/Origin**	**Code**	**Genotype**	**Source/Origin**	**Code**	**Genotype**	**Source/Origin**
G1	0700l	PRI,AARI, Fsd.	G18	NM 92^*^	NIAB, Fsd.	G35	AUM-19	UAF
G2	07002	PRI,AARI, Fsd.	G19	NM 20-21	NIAB, Fsd.	G36	AUM-38	UAF
G3	07003	PRI,AARI, Fsd.	G20	NM 98	NIAB, Fsd.	G37	AUM-27	UAF
G4	07005	PRI,AARI, Fsd.	G21	NM 19-19	NIAB, Fsd.	G38	M-2004	UAF
G5	07006	PRI,AARI, Fsd.	G22	NM-54^*^	NIAB, Fsd.	G39	SM-1	ARS
G6	AZRI-2006	PRI,AARI, Fsd.	G23	Var-6601^*^	NIAB, Fsd.	G40	VC-1482	NIAB/AVRDC
G7	97002	PRI,AARI, Fsd.	G24	NM-28	NIAB, Fsd.	G41	VC-1560^*^	NIAB/AVRDC
G8	97004	PRI,AARI, Fsd.	G25	NM-51	NIAB, Fsd.	G42	VC-1628	NIAB/AVRDC
G9	97006	PRI,AARI, Fsd.	G26	NM 121-25	NIAB, Fsd.	G43	VC-2754	NIAB/AVRDC
G10	97012	PRI,AARI, Fsd.	G27	AUM-6375	UAF	G44	VC-2771	NIAB/AVRDC
G11	97017	PRI,AARI, Fsd.	G28	AUM-18	UAF	G45	VC-2778	NIAB/AVRDC
G12	98001	PRI,AARI, Fsd.	G29	M-2002	UAF	G46	VC-2984B	NIAB/AVRDC
G13	98002	PRI,AARI, Fsd.	G30	M-2006	UAF	G47	VC-3476	NIAB/AVRDC
G14	98005	PRI,AARI, Fsd.	G31	AUM-9	UAF	G48	VC-3902^*^	NIAB/AVRDC
G15	98009	PRI,AARI, Fsd.	G32	AUM-31	UAF	G49	VC-3960	NIAB/AVRDC
G16	NM-2006	NIAB, Fsd.	G33	AUM-24	UAF	G50	VC-6369	NIAB/AVRDC
G17	NM 13-1	NIAB, Fsd.	G34	AUM-28	UAF			

RRI, Fsd., Pulses Research Institute, Faisalabad; UAF, University of Agriculture, Faisalabad, Pakistan; NIAB, Fsd., Nuclear Institute of Agriculture and Biology Faisalabad, Pakistan; ARS, Agricultural Research Station, Mingora, KPK, Pakistan; NIAB/AVRDC, NIAB, Faisalabad/Asian Vegetable and Research Development Centre, Taiwan.

* *Genotypes used for growth habit studies (Khattak et al., 2002b, 2004)*.

#### At screening:

Days to first pod maturity = **D2**

Days to 90% pods maturity = **D3**

Plant height (cm) at first flower initiation = **H1**

Plant height (cm) at 90% pods maturity = **H3**

DDd_2_ was calculated according to the formula outlined by Khattak et al. (2004)

Degree of indetermination (DD) of pod maturity (DDd) from first pod maturity to 90% pods maturity = **DDd**_2_ = D3 - D2/D3 × 100

Degrees of indeterminations of plant height (DDh_1_, DDh_2_, and DDh_3_) were computed by following Khattak et al. (2002b).

Degree of indetermination (DD) of plant height (DDh) from first flower to 90% pods maturity = **DDh**_2_ = H3 - H1/H3 × 100.

#### At final evaluation:

Plant height (cm) at first flower initiation = **H1**

Plant height (cm) at first pod maturity = **H2**

Plant height (cm) at 90% pod maturity = **H3**

Degree of indetermination of plant height from first flower to first pod maturity = **DDh**_1_ = H2 - H1/H2 × 100

Degree of indetermination of plant height from first flower to 90% pods maturity = **DDh**_2_ = H3 - H1/H3 × 100

Degree of indetermination of plant height from first pod maturity to 90% pods maturity = **DDh**_3_ = H3 - H2/H3 × 100

Nodes per plant (no.)

Biological yield per plant (g)

Seed yield per plant (g)

Harvest Index (%) = (Seed yield per plant/Biological yield per plant) × 100 (Reddy, 2004).

Four genotypes with lowest and the highest DDd_2_ and DDh_2_ values were selected. By utilizing the selected four parents two cross combinations were made. Six basic populations (P1, P2, F1, F2, BC1, and BC2) of two crosses were developed (autumn-2009–spring-2010). During final evaluation (autumn 2010), a Complete Randomized Block Design with three replications was exercised. The parents, F1 and back crosses were sown in two rows each, F2 in 20 rows. Twenty random plants were selected from each parent and F1 generation, while plants earmarked from each back cross (BC1 and BC2) and F2 populations were 50 and 100, respectively within a replication. Analysis of variance (ANOVA) and its partitioning was performed according to Steel et al. (1997) by using “Statistix v 8.1” computer software.

### Generation mean and variance analyses

Generation mean analysis was carried out as per Mather and Jinks (1982) by utilizing a computer program supplied by Dr. JW Snape, Cambridge Laboratory, Norwich, for the study of gene action of characters. Mather and Jinks (1982) also outlined the weighted least squares analysis of variance. The same was followed for the experiment comprised of six basic populations. For the purpose a computer programme supplied by Dr. H. S. Pooni, University of Birmingham, UK was utilized. Means and variances of six populations used in the analysis were calculated from individual plants pooled over replications. Characters of the six populations were compared to test the validity of additive-dominance model using Chi-square (χ2) test. Initially simplest model of weighted least square analysis was carried out on generation mean of traits using parameter “m” only. Based on significance of Chi-square value further models md, mdh etc. were adopted. Best selected model taken was the one, with significant values for all the parameters along with non-significant chi-square. Sum of squares for those comparisons were calculated using formula outlined by Little and Hills (1978).

$$\begin{array}{l} \text{SS}=\left(\text{Σ}{\text{c}}_{\text{i}}Yi\right)2∕r\Sigma c_{i}2 \end{array}$$

Where,

SS = sum of squres of comparison

Σ = summation

C_i_ = comparison coefficients

Y_i_ = generation totals

r = replications

Narrow sense heritability (h^2^n.s.) for F2 and infinite (F_∝_) generations were also computed from variance components (D and E) of generation variance analysis

$${\text{h}}_{\left(\text{F2}\right)}^{\text{2}}\text{ = 0}.\text{5D/}(\text{0}.\text{5D + E})$$

(when the simple DE model fitted the data)

$${\text{h}}_{\left(\text{F}\propto \right)}^{\text{2}}\text{ = D/}(\text{D + E})$$

where,

D = additive genetic component

E = environment components

## Results

### Screening of parents and preliminary analysis of variance

Fifty mungbean (Table 1) genotypes were studied for range of variability regarding degree of indetermination of pod maturity from first pod maturity to 90% pods maturity (DDd_2_) and degree of indetermination of plant height from first flower to 90% pods maturity (DDh_2_). The objective was to earmark the parents for hybridization and further studies. Creation of genetic variation and its manipulation plays a decisive role while working out a result oriented breeding strategy (Khattak et al., 2004; Ali et al., 2008). Choice of the parent(s) and selection of trait(s) actually determine the extent of gene action (Kwaye et al., 2008). After collecting data for six various traits at different maturity stages an ordinary analysis of variance (Table 2) was performed. The results signified the existence of variability for the investigated plant characters. Maximum diversity was observed between the parents for the studied traits. Non-significant differences among all the interacting populations were witnessed (Table 3) for the traits, plant height at first flower initiation in both crosses, while for plant height at 90% pods maturity, DDh_1_, DDh_2_, DDh_3_ and seed yield per plant in one cross combination. Both back cross generations (BC1 and BC2) behaved non-significantly for plant height at first flower initiation, DDh_3_, nodes per plant and seed yield per plant in one cross combination. Maximum genetic similarity was observed between the back cross generations (BC1 and BC2) and the F2 population for the traits, plant height at first flower and 90% pods maturity, all degree of indeterminations of plant height, nodes per plant and seed yield.

**Table 2:** **Mean squares of 50 mungbean genotypes for six traits during spring season**.

**Trait**	**D.F.**	**Mean squares**
Days to first pod maturity	49	12.72^**^
Days to 90% pods maturity	49	109.7^**^
DDd_2_	49	53.18^**^
Plant height at first flower initiation	49	17.05^**^
Plant height at 90% pods maturity	49	84.77^**^
DDh_2_	49	78.70^**^

** P < 0.01

**Table 3:** **Mean squares with partitioned generation variances for various traits in two crosses of mungbean**.

	**Cross combination**	**Generations**	**P_1_vs. P_2_**	**P's vs. F_1_**	**BC_1_vs. BC_2_**	**B's vs. F_2_**	**P's, F1 vs. B's, F2**	**Error**
D.F		5	1	1	1	1	1	10
Plant height at first flower initiation	AZRI-2006 × 97006	5.159^**^	16.27^**^	3.170 _NS_	4.060 _NS_	0.004 _NS_	2.300 _NS_	0.832
	NM-2006 × AUM-9	31.35^**^	105.9^**^	22.93^**^	26.47^**^	0.000 _NS_	1.404 _NS_	1.343
Plant height at first pod maturity	AZRI-2006 × 97006	98.30^**^	348.5^**^	24.78^**^	54.80^**^	32.10^**^	31.30^**^	0.943
	NM-2006 × AUM-9	59.25^**^	211.9^**^	23.44^**^	48.53^**^	1.406 _NS_	10.87^**^	1.159
Plant height at 90% pods maturity	AZRI-2006 × 97006	371.6^**^	1291^**^	85.42^**^	210.8^**^	84.94^**^	185.4^**^	2.200
	NM-2006 × AUM-9	156.5^**^	566.8^**^	42.56^**^	153.3^**^	8.332 _NS_	11.57 _NS_	2.850
DDh_1_	AZRI-2006 × 97006	28.39^**^	7.161^**^	17.14^**^	101.3^**^	2.251 _NS_	14.12 _NS_	2.975
	NM-2006 × AUM-9	52.67^**^	32.22^**^	24.51^**^	107.1^**^	0.777 _NS_	98.68^**^	1.479
DDh_2_	AZRI-2006 × 97006	15.26^**^	24.71^**^	4.450 _NS_	19.27^**^	5.894	21.83^**^	1.040
	NM-2006 × AUM-9	45.37^**^	15.57^**^	42.66^**^	156.3^**^	17.50^**^	4.467 _NS_	2.912
DDh_3_	AZRI-2006 × 97006	26.6^**^	29.42^**^	66.21^**^	27.33	2.907 _NS_	7.424	0.762
	NM-2006 × AUM-9	14.51^**^	29.79^**^	32.52^**^	7.358 _NS_	0.006 _NS_	2.123 _NS_	2.248
Nodes per plant	AZRI-2006 × 97006	9.54^**^	39.89^**^	0.462^**^	0.035 _NS_	1.740^**^	0.452^**^	0.075
	NM-2006 × AUM-9	10.21^**^	34.01^**^	0.024 _NS_	2.034^**^	0.066 _NS_	11.02^**^	0.084
Biological yield per plant	AZRI-2006 × 97006	3.458^**^	73.05^**^	18.38^**^	1.025^**^	2.071^**^	0.684^**^	0.045
	NM-2006 × AUM-9	486.45^**^	94.25^**^	15.98^**^	5.640^**^	3.215^**^	5.255^**^	1.602
Harvest index (%)	AZRI-2006 × 97006	3.026^**^	36.51^**^	26.59^**^	14.15^**^	1.056^**^	0.958^**^	0.201
	NM-2006 × AUM-9	3.082^**^	25.61	1.750^**^	2.502^**^	1.035	0.982^*^	0.591
Seed yield per plant	AZRI-2006 × 97006	0.532^*^	0.620^*^	1.887^*^	0.060 _NS_	0.093 _NS_	0.000 _NS_	0.160
	NM-2006 × AUM-9	2.632^**^	8.965^**^	1.811^*^	2.251^**^	0.001 _NS_	0.132^**^	0.003

NS, Non-significant,

* P < 0.05 and

** *P < 0.01*.

*P_1_, Fist parent, P_2_, Second parent, F_1_, First filial generation, BC_1_, Progeny of F_1_ generation after crossing with first parent involved in a cross, BC_2_, Progeny of F_1_ generation after crossing with first parent involved in a cross, F_2_, Second Filial generation*.

**Figure 1:**
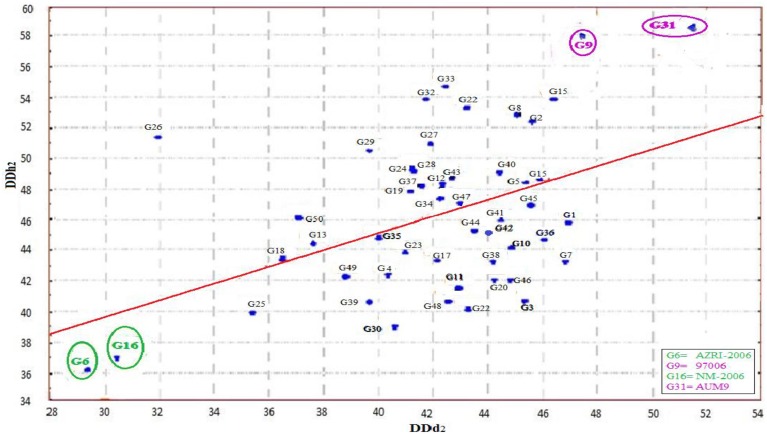
**Scatter plot of DDd_2_ against DDh_2_**.

A scatter plot (Figure 1) was constructed between two variables. In which the variable DDd_2_ was taken at X-axis, while DDh_2_ at Y-axis (Rehman et al., 2009, 2010). The diagram provided the information that two varieties (AZRI-2006 and NM-2006) fall in the zone where the value of DDd_2_ and DDh_2_ was at its minimum (< 38). Accordingly genotype 97006 and AUM-9 had the highest DDd_2_ (>47) and DDh_2_ (>56) values. Most of the genotypes for the trait DDd_2_ ranged from 38 to 48 and that of DDh_2_ from 40 to 50. Thirty eight genotypes were found in that particular patch. A line drawn from the point 38.5 representing generation mean for DDh_2_ on Y-axis, which divided the graph into two portions, each half contains exactly 25 genotypes. Resultantly two varieties (AZRI-2006 and NM-2006) with lowest and the other two genotypes (97006 and AUM-9) with highest DDd_2_ and DDh_2_ values were selected for hybridization and further progenies development. Accordingly after raising the six populations of two crosses, genetic studies were performed.

### Gene action for various traits

A generation mean analysis was used for computing of gene action. The said method is effective for the estimation of additive, dominance and epistatic genetic components, also important for measuring the environmental (E) and G × E interaction (Mather and Jinks, 1982). In addition to other components, additive and dominance genetic effects were the integral part of inheritance for the studied traits, except for plant height at first pod maturity, biological yield per plant and harvest index in NM-2006 × AUM-9 cross, in which dominance component was missing. For plant height at first flower initiation in both crosses and for seed yield per plant in one cross combination only additive and dominance genetic components were important (Table 4) but with a negative dominance. In contrary the said components was also positive for DDh_3_ in case of NM-2006/AUM-9. For the trait, plant height at first pod maturity in the said cross only additive and [i] type epistasis was observed. Additive × additive digenic epistatic interaction was present for seed yield per plant in AZRI-2006/97006 cross. Positive [i] and [j] epistatic digenic interaction was witnessed for DDh_2_. The said interactions were also present for the inheritance of plant height at first and 90% pod maturity, biological yield per plant in one studied cross, with negative value of at least one epistatic component. Similarly [j] and [l] epistatic digenic interactions were observed for DDh_1_ in both the studied greengram crosses and for DDh_2_, DDh_3_ and for biological yield per plant in a single cross. Only negative [i] interaction was noticed for plant height at 90% pods maturity, [j] for harvest index and [l] type interaction for nodes per plant.

**Table 4:** **Estimates of gene effects with standard error and χ^2^ values of the fitted models for plant height and related traits in mungbean**.

**Traits**	**Cross combination**	**m (±SE)**	**[d] (±SE)**	**[h] (±SE)**	**[i] (±SE)**	**[j] (±SE)**	**[l] (±SE)**	**χ2 (d.f)**
Plant height at first flower initiation	AZRI-2006 × 97006	84.5±0.8	6.15±0.3	−2.80±0.5				1.60 (3)
	NM-2006 × AUM-9	24.8±0.2	4.21±0.2	−3.39±0.5				0.01 (3)
Plant height at first pod maturity	AZRI-2006 × 97006	38.1±1.2	19.7±0.4	−8.92±1.7	−8.79±1.29	−13.3±1.0		3.80 (1)
	NM-2006 × AUM-9	35.3±0.4	5.91±0.4		3.33±0.56			0.10 (3)
Plant height at 90% pods maturity	AZRI-2006 × 97006	59.6±1.3	45.6±0.3	−19.2±1.6	−17.4±1.33	−22.9±1.2		0.80 (1)
	NM-2006 × AUM-9	59.9±1.6	9.80±0.5	−11.9±2.0	−7.36±1.70			0.10 (2)
DDh_1_	AZRI-2006 × 97006	45.4±0.7	10.8±0.7	8.79±3.6		−7.30±1.8	−8.85±3.6	0.20 (1)
	NM-2006 × AUM-9	37.5±0.4	2.32±0.3	−17.9±1.7		6.10±0.9	21.4±1.7	1.10 (1)
DDh_2_	AZRI-2006 × 97006	53.9±0.6	8.04±0.6	31.5±2.9		−12.8±1.6	−31.8±2.9	0.30 (1)
	NM-2006 × AUM-9	47.2±1.2	0.96±0.4	11.5±1.5	6.84±1.27	9.50±1.1		3.00 (1)
DDh_3_	AZRI-2006 × 97006	32.2±0.6	4.12±0.6	16.9±2.9		−4.90±1.5	−17.6±2.9	0.10 (1)
	NM-2006 × AUM-9	27.3±0.3	2.27±0.3	4.08±0.6				0.01 (3)
Nodes per plant	AZRI-2006 × 97006	7.68±0.4	2.06±0.1	3.65±0.5			−0.74±0.1	0.05 (2)
	NM-2006 × AUM-9	6.48±0.3	2.56±0.4	5.50±0.9			−1.97±0.1	0.04 (2)
Biological yield per plant	AZRI-2006 × 97006	61.5±1.1	13.7±0.7	9.25±0.5		−10.5±1.1	−5.84±0.4	2.51 (2)
	NM-2006 × AUM-9	58.8±1.8	7.58±0.5		5.58±0.21	−8.99±1.2		1.23 (2)
Harvest index (%)	AZRI-2006 × 97006	21.6±0.5	3.56±0.2	11.6±1.4		4.15±0.4		0.30 (2)
	NM-2006 × AUM-9	23.5±0.4	6.14±0.9			−4.84±0.4		2.41 (3)
Seed yield per plant	AZRI-2006 × 97006	13.2±0.2	0.31±0.1	1.60±0.3	0.56±0.20			0.70 (2)
	NM-2006 × AUM-9	13.8±0.1	1.20±0.1	−0.96±0.1				0.06 (3)

*m, mean; [d], additive; [h], dominance; [i], additive × additive; [j], additive × dominance; [l], dominance × dominance*.

### Genetic component of variance and narrow sense heritability estimates

Generation variance analysis can partition the total variation into different components i.e., additive (D), dominance (H), environmental (E) and interaction (F). Non-segregating (e.g., pure lines, inbred lines, F1 etc.) and segregating (e.g., backcrosses and F2) populations were utilized for the estimation of genetic and environmental component of variance in the present study (Table 5). A non-significant χ^2^ value was observed with two parameters [DE] model only. Additive component of variance (D) was much higher than the corresponding environmental (E) variance in all the studied traits, except for seed yield per plant. The values of additive and environmental components ranged from 3.262–206.1 and 0.237–31.90, respectively for AZRI-2006 × 97006 cross. The values for the same parameters ranged from 0.132–127.6 and 0.089–14.03, respectively in NM-2006/AUM-9.

**Table 5:** **Best fit model following weighted analysis of components of variation and narrow sense heritability estimates in two crosses of mungbean**.

**Traits**	**Cross combination**	**Variance components**		**χ^2^(4df)**	**Heritability (%age)**	
		**D (±SE)**	**E (±SE)**		**h^2^_(*F*2)_**	**h^2^_(*F*∝)_**
Plant height at first flower initiation	AZRI-2006 × 97006	38.70±5.35	6.137±0.90	3.723	75.9	86.3
	NM-2006 × AUM-9	36.07±4.49	4.336±0.63	1.123	80.6	90.3
Plant height at first pod maturity	AZRI-2006 × 97006	65.19±9.02	10.36±1.52	1.680	75.8	86.3
	NM-2006 × AUM-9	93.32±11.1	9.846±1.45	3.531	82.6	90.5
Plant height at 90% pods maturity	AZRI-2006 × 97006	119.6±12.1	6.433±0.95	1.317	90.3	94.9
	NM-2006 × AUM-9	127.6±19.1	14.03±2.07	1.428	86.0	92.5
DDh_1_	AZRI-2006 × 97006	206.1±28.2	31.90±4.68	2.461	76.4	86.6
	NM-2006 × AUM-9	44.62±6.58	8.176±1.19	1.920	73.2	84.5
DDh_2_	AZRI-2006 × 97006	174.2±20.7	18.43±2.72	1.053	82.5	90.4
	NM-2006 × AUM-9	90.78±10.4	8.423±1.24	2.416	84.3	91.5
DDh_3_	AZRI-2006 × 97006	123.4±18.5	23.52±3.44	4.924	72.4	84.0
	NM-2006 × AUM-9	39.96±5.63	6.614±0.97	0.509	75.1	85.8
Node per plant	AZRI-2006 × 97006	18.12±2.93	6.251±0.87	0.957	78.0	82.1
	NM-2006 × AUM-9	6.547±0.35	0.420±0.85	1.351	71.2	82.4
Biological yield per plant	AZRI-2006 × 97006	202.5±24.1	36.1±3.58	3.042	69.7	82.6
	NM-2006 × AUM-9	31.92±4.14	5.16±0.91	1.122	68.2	64.9
Harvest index (%)	AZRI-2006 × 97006	26.98±3.19	2.452±0.35	0.645	70.4	83.1
	NM-2006 × AUM-9	33.85±4.84	3.150±0.84	1.452	73.4	83.4
Seed yield per plant	AZRI-2006 × 97006	3.262±0.32	0.137±0.021	0.251	92.6	96.0
	NM-2006 × AUM-9	1.132±0.12	0.089±0.01	5.481	86.4	92.7

*D, additive variance; E, environmental variance; h^2^(_F2_), narrow sense heritability F2 generation and h^2^(_F∝_), narrow sense heritability F infinite generation*.

Narrow sense heritability F2 generation (h${}_{\text{F}2}^{2}$) was minimum for the trait; biological yield per plant and maximum for seed yield per plant with respective values 69.7 and 92.6 (Table 5), for AZRI-2006 × 97006 cross. The said heritability estimate was minimum for biological yield per plant (68.2) and maximum for seed yield per plant (86.4) in NM-2006 × AUM-9 cross. Infinite generation narrow sense heritability (h${}_{\text{F}\propto }^{2}$) was minimum for biological yield per plant and maximum for seed yield per plant, surpassing plant height at 90% pods maturity with respective values 69.7, 96.0, and 94.9, for AZRI-2006 × 97006 cross. Similarly for NM-2006 × AUM-9 cross combination minimum narrow sense heritability for infinite generation was witnessed for biological yield per plant (68.2) and maximum for seed yield per plant (92.7).

## Discussion

Determinate plant growth in grain legumes could facilitate effective assimilates partitioning and dwarfism could bring reduction in lodging. Modification in leaf size, structure and LAI could improve the light interception efficiency and ultimately the harvest index of the crop. Thereby modified crop architecture could play a pivotal role while improving the adoptability of the grain legumes to wider environmental conditions and to increase the seed yield and its stability (Huyghe, 1998). Both crop and plant architecture fluctuates with a change in plant growth and dry matter accumulation. With an efficient breeding strategy crop architect could be modified. Observations were made regarding the influential impact of plant stem growth on various agronomical characters. For instance determinate plants remained dwarf, resist lodging and have lower lowest-pod heights and maximum main stem branches in comparison to indeterminate genotypes at similar maturity (Ouattara and Weaver, 1994; Robinson and Wilcox, 1998; Kilgore-Norquest and Sneller, 2000). The transition from vegetative to reproductive phase at the shoot apical meristem is controlled by the interaction of positive and negative regulators and triggered through genes (Benlloch et al., 2007). The same control the floral primordia development at the summit of an inflorescence apex. Therefore, an understanding about the nature and extent of such gene/genes is pre-requisite for launching an effective breeding plan. But for the manipulation of this aspect, availability of a diversified genetic stock with both the extremes of characters is necessary. Subsequently intermating the genetically diverse parents could enhance the genetic variance by supporting more than one optimum in a plant population (Manifesto et al., 2001). The scatter plot revealed that AZRI-2006 and NM-2006 were the most synchronous maturing (< DD_2_) and determinate type (< DDh_2_) mungbean varieties. Lower estimates of degree of indeterminations in the approved varieties could serve as an eye opener for the breeders, regarding the possibility of reduction/elimination of the phenomenon of indeterminate growth habit in mungbean through genetic means. The same feature of the said varieties could be manipulated in the future breeding programmes. The accession 97006 and AUM-9 by virtue of their asynchronous and twinning growth habit could serve as potential parent for crop growth related studies.

The significance of only D and E component in genetic variance analysis exhibited the pre-pondrance of additive and environmental components. The environment could fluctuate the plant height and its degree of indeterminations but with nominal impact. Genotypic × environmental interaction and its involvement in the inheritance were also reported by Deswal et al. (1996) in wheat and Khattak et al. (2002a) in mungbean. Additive genetic variance played a vital role in the inheritance of investigated traits, might be due to high narrow sense heritability (h${}_{\text{F}2}^{2}$ and h${}_{\text{F}\propto }^{2}$) estimates. The same reiterated the involvement of few major genes and similar genetic effects and likelihood of genetic improvement of all the studied traits. Any protective measure that could minimize the experimental error may improve the estimate of heritability of a trait (Fehr, 1987). Khattak et al. (2002b) also computed high narrow and broad sense heritability estimates for DDh_2_, They further explained that better response to selection is possible for the development of mungbean genotypes with minimum increase in plant height during post-flowering development. Engagements of epistasis for most of the traits in the present study reaffirm the availability of sufficient genetic variation. A negative dominance for plant height approaching reproductive phase and seed yield per plant specified the involvement of sufficient negative genes. Due to the accumulation of negative genes selection for dwarf type plants at blooming phase with higher seed yield could be postponed to later generation until the accretion of favorable genes. However, the dominance in case of DDh_3_ is toward lower degree of indetermination, therefore for the same selection could be practice in early segregating generation. So bulk, pedigree or single seed descendent method of selection could be opted. Presence of higher magnitude of additive gene action for plant height was reported by Sharma et al. (2008) in peas and Verma et al. (2007) in barley. Additive and dominance gene action governed the inheritance of most traits in long bean (Rahman and Saad, 2000) and for plant height at first and 90% pods maturity, DDh_1_, DDh_2_, and DDh_3_ in mungbean (Khattak et al., 2002b). Duplicate epistasis was observed for the inheritance of plant height in mungbean (Ajmal et al., 2007; Khodambashi et al., 2012). Involvement of non-additive gene action for the inheritance of seed yield was reported by Kunkaew et al. (2007) in adzuki bean and Sujatha and Kajjidoni (2013) in greengram and additive, dominance and non-allelic interactions for plant height have also been document in greengram (Singh et al., 2007). Various negative digenic epistatic interactions witnessed in the present study reflect the non-availability of favorable genes. Non-additive gene action for seed yield in cowpea was also observed (Dijee et al., 2000). The inheritance of plant height is governed by non-additive gene action in mungbean (Tiwari et al., 1993) in sweet sorghum (Sankarapandian et al., 1994) and also by duplicate epistasis (Khodambashi et al., 2012) in greengram. Evidence of di-genic non-allelic interactions accompanied with additive and dominance components have also been documented for the inheritance of plant height and degree of indetermination in green gram (Ram, 1997). Additive and non-additive gene actions control the inheritance of plant height in mungbean (Singh et al., 2007) and harvest index in wheat (Chand and Dawa, 1996). Several geneticists witnessed the existence of epistasis in the inheritance of quantitative character in different crops (Pensuk et al., 2004; Bnejd and El- Gazzah, 2008, 2010; Shashikumar et al., 2010). Accumulation and clustering of interacting sets of genes with additive influence could provide a path for the genetic improvement of quantitative traits. Digenic epistatic interactions governed the inheritance of most of the studied traits. For the exploitation of such epistasis, development of multiple crosses and rising of large segregating populations followed be inter se mating of desired segregants could help in piling-up the frequency of additive genes. Intermating or recurrent selection would be followed for genetic enhancement of grain yield in mungbean (Payasi et al., 2010). The same will also favor the development of potential transgressive segregants and breakage of unwanted linkages. After inter mating the desired segregants, one or two cycles of recurrent selection and each cycle of selection followed by selfing to one generation while deferring the final selection to later filial generation could serve as a good promising method for the elimination of negative genes and development of dwarf and determinate type mungbean lines with high harvest index. Singh and Pawar (1990) also suggested recurrent selection procedures for the exploitation of non-additive genetic variability.

## Conclusion

The choice of most suitable parents, careful planning of crosses, selection and intermating of desired segregants could be the key factors for devising a work oriented breeding programme for restricting the indeterminate growth in mungbean.

## Conflict of interest statement

The authors declare that the research was conducted in the absence of any commercial or financial relationships that could be construed as a potential conflict of interest.
